# Widespread introgression in deep-sea hydrothermal vent mussels

**DOI:** 10.1186/s12862-016-0862-2

**Published:** 2017-01-13

**Authors:** Corinna Breusing, Robert C. Vrijenhoek, Thorsten B. H. Reusch

**Affiliations:** 1 GEOMAR Helmholtz Centre for Ocean Research Kiel, Evolutionary Ecology of Marine Fishes, Düsternbrooker Weg 20, 24105 Kiel, Germany; 2 Monterey Bay Aquarium Research Institute, 7700 Sandholdt Road, Moss Landing, CA 95039 USA

**Keywords:** *Bathymodiolus*, Introgressive hybridization, Hybrid zone models, Single nucleotide polymorphisms

## Abstract

**Background:**

The analysis of hybrid zones is crucial for gaining a mechanistic understanding of the process of speciation and the maintenance of species boundaries. Hybrid zones have been studied intensively in terrestrial and shallow-water ecosystems, but very little is known about their occurrence in deep-sea environments. Here we used diagnostic, single nucleotide polymorphisms in combination with one mitochondrial gene to re-examine prior hypotheses about a contact zone involving deep-sea hydrothermal vent mussels, *Bathymodiolus azoricus* and *B. puteoserpentis*, living along the Mid-Atlantic Ridge.

**Results:**

Admixture was found to be asymmetric with respect to the parental species, while introgression was more widespread geographically than previously recognized. Admixed individuals with a majority of alleles from one of the parental species were most frequent in habitats corresponding to that species. Mussels found at a geographically intermediate vent field constituted a genetically mixed population that showed no evidence for hybrid incompatibilities, a finding that does not support a previously inferred tension zone model.

**Conclusions:**

Our analyses indicate that *B. azoricus* and *B. puteoserpentis* hybridize introgressively across a large geographic area without evidence for general hybrid incompatibilities. While these findings shed new light onto the genetic structure of this hybrid zone, many aspects about its nature still remain obscure. Our study sets a baseline for further research that should primarily focus on the acquisition of additional mussel samples and environmental data, a detailed exploration of vent areas and hidden populations as well as genomic analyses in both mussel hosts and their bacterial symbionts.

**Electronic supplementary material:**

The online version of this article (doi:10.1186/s12862-016-0862-2) contains supplementary material, which is available to authorized users.

## Background

Hybrid zones, regions where genetically distinct species interbreed to form genotypes of mixed origin, have achieved considerable interest in evolutionary research. Such zones of interbreeding can provide significant insights into the mechanisms that underlie speciation and the integrity of species [1, 2]. Interspecific hybridization in animals has traditionally been considered as a mechanism causing genetic swamping and extinction of species (reverse speciation) [3–7], but increasing evidence stresses its importance as a process that promotes adaptive variation and the evolution of biodiversity [1, 2, 4, 8]. These destructive and creative mechanisms represent relatively extreme outcomes of hybridization, which typically acts as selective filter to between-species gene flow, thereby leading to complex pictures of differential introgression across the genome [9]. While selectively neutral loci will introgress freely across the hybrid zone, loci involved in reproductive isolation or local adaptation are likely to show restricted introgression. The identification of such barrier genes is crucial to understand the genomic architecture of species boundaries. Depending on the spatial scale as well as the environmental, historical and geographic context, hybrid zones vary in their genetic structures. In many cases interspecific mating is spatially confined to a narrow region that is bordered by pure populations of the interbreeding lineages. Such tension zones [3] are maintained by a dynamic equilibrium between immigration of parental species and selection against hybrids, a scenario that results in concordant step clines in allelic frequencies. If the distributions of the hybridizing taxa are discontinuous, hybrid zones are commonly composed of multiple population patches that represent individual local contact areas between the hybridizing taxa [9]. In contrast to tension zones, patchy hybrid zones often extend across large geographic regions and either reflect colonization history (mottled hybrid zones) or ecological segregation (mosaic hybrid zones) of the interbreeding species. Under this scenario admixed individuals comprise a diverse “swarm” of genotypes that can facilitate local adaptation and potentially lead to hybrid speciation [1].

**Fig. 1:**
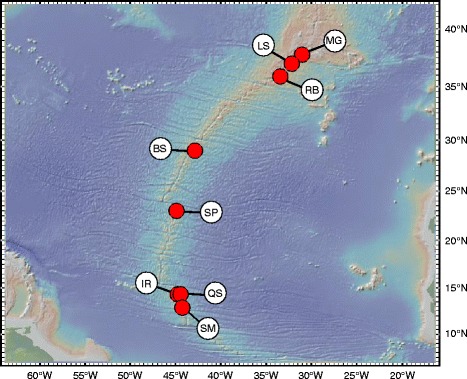
Sampling area of *Bathymodiolus* mussels along the Mid-Atlantic Ridge. MG = Menez Gwen, LS = Lucky Strike, RB = Rainbow, BS = Broken Spur, SP = Snake Pit, IR = Irina, QS = Quest, SM = Semenov. Adapted from [28] with permission from ELSEVIER

Although most hybrid zone research has focused on terrestrial, freshwater and coastal ecosystems, three examples of hybridization and intergradation were described from deep-sea hydrothermal vent environments [10–13]. Unlike the open ocean, vent habitats are geomorphologically constrained and distributed relatively linearly along the global mid-ocean ridge system, sporadically in back-arc spreading centres or on volcanically active seamounts [14]. Offsets in the axis of mid-ocean ridges and the isolated nature of back-arc basins can restrict the flow of deep-water currents disrupting the dispersal of vent larvae. This, in turn, may produce various degrees of geographic subdivision in vent animals [15, 16]. These circumstances are likely to influence the opportunities and outcomes of hybridization in deep-sea vent species. Morphologically and genetically divergent bathymodiolin mussels native to the Mid-Atlantic Ridge (MAR) (Fig. 1) provided the first example of a hybrid zone at deep-sea hydrothermal vents [10, 11, 17–19]. *Bathymodiolus azoricus* inhabits shallower vents (813–2251 m) clustered near the Azorean Triple Junction [18], whereas *Bathymodiolus puteoserpentis* inhabits deeper vents (2432–3480 m) distributed along the central MAR [17, 18]. These mussels hybridize at the latitudinally intermediate Broken Spur (BS) vent field that does not appear to be particularly suitable for either species [10, 11]. Elsewhere the two ecologically similar parental species are remarkably abundant. They both depend nutritionally on chemosynthetic gammaproteobacterial endosymbionts that are capable of oxidizing methane, hydrogen sulphide, and hydrogen present in the vent effluents [20]. They are gonochoristic with periodic, external fertilization and long-lived planktotrophic larvae, although instances of protandric hermaphroditism have been reported in *B. azoricus* [21–24]. Spawning of *B. azoricus* takes place between January and March [24], but the reproductive season of *B. puteoserpentis* is unknown.

Won et al. [11] found that mussels from the BS hybrid zone exhibited significant cytonuclear disequilibrium between mitochondrial NADH dehydrogenase subunit 4 (*ND4*) haplotypes and leucine-aminopeptidase (*Lap*) allozymes. Parental genotypes greatly surpassed the frequency of recombinants, indicating limited reproduction and self-recruitment at the environmentally stressful BS locality. Concordant step-clines across a number of allozyme loci suggested that introgression into the parental ranges was limited. Together these patterns were interpreted as evidence for a tension zone maintained primarily by immigration of parental species and possibly hybrid unfitness. Faure et al. [25] developed a number of nuclear DNA markers to investigate the origin of the hybrid zone. They concluded that *B. azoricus* and *B. puteoserpentis* split as recently as 0.76 million years ago. The coalescent history of intragenic recombination between semi-diagnostic *GF1B* alleles led these authors to surmise that secondary intergradation probably followed a brief period of allopatric divergence. Although biogeography can be useful in the interpretation of genetic patterns [26], a lack of multiple, diagnostic, nuclear markers has so far limited researchers’ abilities to identify the nature of the hybrids and to assess the accuracy of the tension zone model due to the difficulty of distinguishing introgressive hybridization from incomplete lineage sorting (retention of ancestral polymorphisms)—a common problem involving species that have split very recently or have retained very large effective population sizes [27].

To remedy these limitations, the present study builds upon a recent development. Breusing et al. [28] designed a panel of single nucleotide polymorphisms (SNPs) to study gene flow across the known ranges of *B. azoricus* and *B. puteoserpentis*. For the present study, we used a diagnostic subset of these SNP markers and the mitochondrial *ND4* gene (i) to identify the geographical range of hybridization along the MAR axis, (ii) to thoroughly determine different classes of hybrids, and (iii) to find evidence for or against the tension zone model.

## Methods

### Sample collection and molecular analyses

Mussel samples were obtained from eight localities (Fig. 1) during research expeditions conducted between 1997 and 2013 along the northern MAR (37°50.7′N to 13°30.8′N). For geographic coordinates, bathymetric depths, and sample sizes see Additional file 1: Table S1. To accurately assess hybridization between *B. azoricus* and *B. puteoserpentis*, we used a subset of 18 species-diagnostic markers from a starting panel that included 90 SNPs [28]. Loci were defined as diagnostic, if the two SNP alleles were reciprocally abundant with ≥ 95% in the most distant geographical populations MG (*B. azoricus*) and SM (*B. puteoserpentis*) (Additional file 2: Tables S2 and S3). In addition to the 18 SNP markers, we included the mitochondrial *ND4* locus that was shown to be diagnostic for the two lineages [10, 11]. Limiting our analyses to highly differentiated markers allowed us to distinguish incomplete lineage sorting from introgression [27] and to obtain reliable estimates of hybrid indexes to categorize genotypes into hybrid classes [29]. *ND4* sequences, haplotype data and SNP genotypes for all mussel specimens were taken from Breusing et al. [28], where full details about the molecular analyses are available. Further information about individual genotypes, primer sequences and putative gene functions are given in Additional file 2: Tables S3 and S4.

### Simulation of artificial hybrid populations

We applied STRUCTURE v2.3.4 [30], INTROGRESS (R v3.2.0) [31–33] and NEWHYBRIDS v1.1b [34] to identify the degree of admixture and introgression across the deep-sea hybrid zone. To assess the power of these population genetic programs to resolve hybridization, we modelled hybrid and pure genotypes in HYBRIDLAB v1.1 [35], using the MG and SM samples as parental populations. Simulations were performed as described in the manual with sample sizes of 40 individuals per population. Model runs for STRUCTURE, INTROGRESS and NEWHYBRIDS were done as for the real samples described in “*Population structure and admixture in the real data set*”.

For STRUCTURE, we identified the highest and lowest average *q*-values in the simulated parental populations of *B. azoricus* (0.05) and *B. puteoserpentis* (0.92) to set these as thresholds for hybrid identification. As STRUCTURE *q*-values do not consider interspecific heterozygosities and are therefore not suitable for distinguishing between different hybrid genotypes, we did not attempt to further classify potential hybrid individuals with this program. For INTROGRESS, hybrid categorizations essentially followed the classification scheme by Milne & Abott [36], considering that some hybrid types cannot be distinguished due to identical ranges of hybrid indices (*h*) and interspecific heterozygosities (*IH*). Reference values of *h* and *IH* were determined for artificial hybrid and parental types and subsequently adjusted as given in Table 1. For NEWHYBRIDS, we defined 12 genotype categories (Table 1) to allow detection of backcross hybrids and distinguish those from pure individuals. The mean genotype probability across five replicate runs was used to assign individuals to the most appropriate hybrid class. As different backcross categories could not be distinguished from each other (see above) and to make the NEWHYBRIDS output comparable to the other programs, backcross hybrid categories for each species were lumped into one class and their probabilities were summarized.

**Table 1:** Genotype categories as used by the NEWHYBRIDS and INROGRESS programs based on simulations in HYBRIDLAB

Genotype category	NEWHYBRIDS				INTROGRESS	
	AA	Aa	aA	aa	*IH*	*h*
*B. azoricus*	1.00000	0.00000	0.00000	0.00000	0.00–0.11	0.00–0.03
BC1 *azoricus*	0.50000	0.25000	0.25000	0.00000	0.00–0.88	0.04–0.27
BC2 *azoricus*	0.75000	0.12500	0.12500	0.00000		
BC3 *azoricus*	0.87500	0.06250	0.06250	0.00000		
BC4 *azoricus*	0.93750	0.03125	0.03125	0.00000		
F_1_	0.00000	0.50000	0.50000	0.00000	0.89–1.00	0.45–0.53
F_2–4_	0.25000	0.25000	0.25000	0.25000	0.00–0.88	0.28–0.79
BC1 *puteoserpentis*	0.00000	0.25000	0.25000	0.50000	0.00–0.88	0.80–0.93
BC2 *puteoserpentis*	0.00000	0.12500	0.12500	0.75000		
BC3 *puteoserpentis*	0.00000	0.06250	0.06250	0.87500		
BC4 *puteoserpentis*	0.00000	0.03125	0.03125	0.93750		
*B. puteoserpentis*	0.00000	0.00000	0.00000	1.00000	0.00–0.17	0.94–1.00

For NEWHYBRIDS categories are based on genotype frequency proportions, where *A* denotes the *B. azoricus*-specific allele and *a* denotes the *B. puteoserpentis*-specific allele. For INTROGRESS categories are based on interspecific heterozygosities (*IH*) and hybrid indices (*h*)

### Population structure and admixture in the real data set

STRUCTURE was used to determine population genetic structuring and patterns of hybridization between *B. azoricus* and *B. puteoserpentis* along the MAR. Simulations based on the SNP data were run according to the admixture model with correlated allele frequencies [37], testing up to 10 genetic clusters *K* with 10 replicates. Model runs involved 10^7^ iterations after a de-memorization period of 10^6^. For identifying the most likely value of *K* we applied the Δ*K* correction as described in Evanno et al. [38] and inspected bar plots for biologically reasonable structuring [39]. STRUCTURE graphics were produced with “bar_plotter.rb” (http://evolution.unibas.ch/salzburger/software.htm). Haplotype networks for population structure analyses of the mitochondrial *ND4* locus were created with NETWORK v4.6.1.2 (www.fluxus-engineering.com; [40–42]) as in Breusing et al. [43]. To assess the level of reproductive isolation and infer evolutionary processes structuring the hybrid zone we calculated Hardy-Weinberg equilibrium (HWE) and linkage disequilibrium (LD) in GENEPOP v4.3 [44] using 10,000 dememorization steps, a batch length of 10,000 and a batch number of 1000. HWE tests were done with the enumeration method. Corrections for multiple comparisons (*α* = 0.05) were performed with the Benjamini-Yekutieli False Discovery Rate (BY FDR) procedure [45], which has been found to produce biologically more meaningful results than comparable methods [46].

To estimate individual hybrid indices *h* and interspecific heterozygosities *IH* based on genotypic information at the 18 diagnostic SNP markers, we used the maximum likelihood approach implemented in the R package INTROGRESS, defining MG and SM as parental populations of *B. azoricus* and *B. puteoserpentis*, respectively. To support the findings of the INTROGRESS program, we performed additional simulations in NEWHYBRIDS. Runs were replicated five times with different seed values, using 100,000 iterations of the MCMC chain and a burnin of 10,000.

## Results

### Detection of hybrids and genotype classes in the artificial population data set

Simulations of hybrid categories confirmed that different F_x_ generation hybrids and parental genotypes were easily distinguishable from each other using the 18 diagnostic SNP markers, as all programs showed a very high assignment certainty for individuals from these classes (Additional file 3: Table S5; Additional file 4: Figure S1). Although STRUCTURE could not be used to distinguish various hybrid generations, it assigned 100% of the F-type individuals correctly as hybrids. Likewise, all *B. azoricus* and *B. puteoserpentis* individuals were identified accurately by this program. INTROGRESS and NEWHYBRIDS were applied to further differentiate between distinct hybrid genotypes. While INTROGRESS seemed to group all F_1_, F_2–4_ and pure individuals into the appropriate categories, NEWHYBRIDS showed a misidentification rate of ≤ 5% for F_x_ generation hybrids and ≤ 10% for parental species. Probabilities for correct assignments of both parental and F_x_ genotypes were generally high (*B. puteoserpentis*: 0.639; *B. azoricus*: 0.690; F_1_: 0.997; F_2–4_: 0.964 − 0.984).

By contrast, the identification of backcross genotypes was less reliable, as many individuals were misclassified as either F_2–4_ hybrids (BC1, BC2) or pure species (BC3, BC4). STRUCTURE categorized up to 87.5% incorrectly as parental genotypes given that *q*-values largely overlapped between these classes (Additional file 5: Figure S2). Similarly, INTROGRESS misidentified up to 77.5% as F_2–4_ hybrids or parental species. NEWHYBRIDS assignments appeared to be more accurate and became only very uncertain for later generation backcrosses, where between 25 and 40% were wrongly assigned as pure individuals (Additional file 3: Table S5; Additional file 4: Figure S1). Probabilities for the correct classification were in a similar range as for the other genotype categories (0.772 − 0.991). Since (backcross) hybrids were much more frequently misidentified as parental species than vice versa, the HYBRIDLAB simulations suggest that the amount of genetic admixture in our real population data set could be significantly underestimated. Unfortunately, such wrong assignments might be difficult to detect, as the NEWHYBRIDS probabilities for misidentified individuals were usually only slightly lower than for correct classifications (Additional file 3: Table S5; Additional file 4: Figure S1).

### Genetic structure and extent of hybridization in the real population data set

**Fig. 2:**
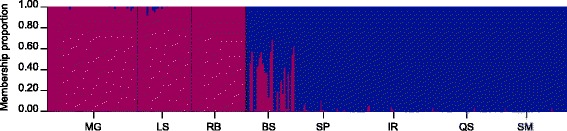
Inferred genetic structure for the eight *Bathymodiolus* sampling localities based on the 18 diagnostic SNP markers. The graph confirms the existence of two mussel species (*B. azoricus* in *red*, *B. puteoserpentis* in *blue*) that are interbreeding in an asymmetric way along the Mid-Atlantic Ridge, as shown by the presence of individuals (*vertical lines*) with mixed ancestry (*q*-values between 0.05 and 0.92). MG = Menez Gwen, LS = Lucky Strike, RB = Rainbow, BS = Broken Spur, SP = Snake Pit, IR = Irina, QS = Quest, SM = Semenov. Adapted from [28] with permission from ELSEVIER

The STRUCTURE analyses confirmed the presence of two genetic groups corresponding with the recognized species, *B. azoricus* and *B. puteoserpentis* (Fig. 2). At the northern vents MG, LS and RB populations consisted exclusively of parental *B. azoricus* genotypes (MG, RB) or comprised a mixture of mostly pure *B. azoricus* and a low amount of hybrids (LS; 6.7%). By contrast, at the southern vents SP, IR, QS and SM populations contained only *B. puteoserpentis* individuals (IR, QS, SM) or were a mixture of pure *B. puteoserpentis* and a few hybrid mussels (SP; 3.3%). At the intermediate BS locality 60% of mussels seemed to be hybrids, whereas the rest of the individuals were classified as pure *B. puteoserpentis*.

**Fig. 3:**
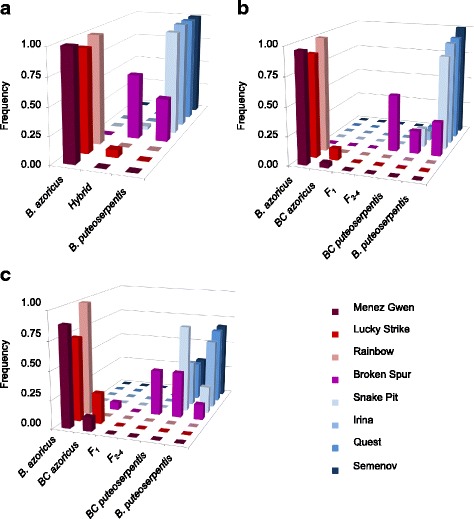
Frequencies of hybrid and parental genotypes in the real population data set based on **a** STRUCTURE, **b** INTROGRESS and **c** NEWHYBRIDS

To further resolve the extent of introgression and the nature of the hybrids we used maximum likelihood and Bayesian inference methods in the programs INTROGRESS and NEWHYBRIDS. Although these analyses confirmed the asymmetric genotype distribution pattern that was already indicated by the STRUCTURE simulations, they identified additional hybrid individuals at various locations of the MAR, implying a more widespread occurrence of introgression (Fig. 3; Table 2). While backcrosses to *B. azoricus* were detected at MG (4.0 − 12.0%) and LS (10.0 − 26.7%), backcrosses to *B. puteoserpentis* were detected at SP (16.7 − 76.7%), IR (8.3 − 39.6%), QS (6.7 − 33.3%) and SM (0.0 − 32.5%). At the central vent BS 40 − 50% of the individuals were categorized as multi-generation hybrids (F_2_ to F_4_) with a similar allelic contribution from both parental species (Fig. 3; Table 2). The remaining individuals were classified as pure *B. puteoserpentis* (13.3 − 30.0%) and backcrosses thereof (20.0 − 40.0%), whereas no *B. azoricus* were found at this locality. In contrast to the INTROGRESS program, NEWHYBRIDS identified a few backcrosses to *B. azoricus* (6.7%) at BS, although these might be misclassified F-generation hybrids, as indicated by results from the artificial population data set (Additional file 3: Table S5). F_1_ hybrids were completely absent from all locations. In general, NEWHYBRIDS assigned many more individuals as hybrids than did any of the other programs, while STRUCTURE was most conservative and identified the majority of individuals as parental species (Table 2).

**Table 2:** Genotype frequencies in the eight sampled mussel populations as inferred by STRUCTURE, INTROGRESS and NEWHYBRIDS

	STRUCTURE	INTROGRESS	NEWHYBRIDS	
	Frequency	Frequency	Frequency	Probability (N)
Menez Gwen				
*B. azoricus*	1.000	0.960	0.880	0.688 (44)
BC *azoricus*	0.000	0.040	0.120	0.741 (6)
F_1_		0.000	0.000	NA
F_2–4_		0.000	0.000	NA
BC *puteoserpentis*		0.000	0.000	NA
*B. puteoserpentis*	0.000	0.000	0.000	NA
Lucky Strike				
*B. azoricus*	0.933	0.900	0.733	0.692 (22)
BC *azoricus*	0.067	0.100	0.267	0.794 (8)
F_1_		0.000	0.000	NA
F_2–4_		0.000	0.000	NA
BC *puteoserpentis*		0.000	0.000	NA
*B. puteoserpentis*	0.000	0.000	0.000	NA
Rainbow				
*B. azoricus*	1.000	1.000	1.000	0.673 (30)
BC *azoricus*	0.000	0.000	0.000	NA
F_1_		0.000	0.000	NA
F_2–4_		0.000	0.000	NA
BC *puteoserpentis*		0.000	0.000	NA
*B. puteoserpentis*	0.000	0.000	0.000	NA
Broken Spur				
*B. azoricus*	0.000	0.000	0.000	NA
BC *azoricus*	0.600	0.000	0.067	0.751 (2)
F_1_		0.000	0.000	NA
F_2–4_		0.500	0.400	0.966 (12)
BC *puteoserpentis*		0.200	0.400	0.814 (12)
*B. puteoserpentis*	0.400	0.300	0.133	0.639 (4)
Snake Pit				
*B. azoricus*	0.000	0.000	0.000	NA
BC *azoricus*	0.033	0.000	0.000	NA
F_1_		0.000	0.000	NA
F_2–4_		0.000	0.000	NA
BC *puteoserpentis*		0.167	0.767	0.739 (23)
*B. puteoserpentis*	0.967	0.833	0.233	0.637 (7)
Irina				
*B. azoricus*	0.000	0.000	0.000	NA
BC *azoricus*	0.000	0.000	0.000	NA
F_1_		0.000	0.000	NA
F_2–4_		0.000	0.000	NA
BC *puteoserpentis*		0.083	0.396	0.756 (19)
*B. puteoserpentis*	1.000	0.917	0.604	0.640 (29)
Quest				
*B. azoricus*	0.000	0.000	0.000	NA
BC *azoricus*	0.000	0.000	0.000	NA
F_1_		0.000	0.000	NA
F_2–4_		0.000	0.000	NA
BC *puteoserpentis*		0.067	0.333	0.667 (10)
*B. puteoserpentis*	1.000	0.933	0.667	0.651 (20)
Semenov				
*B. azoricus*	0.000	0.000	0.000	NA
BC *azoricus*	0.000	0.000	0.000	NA
F_1_		0.000	0.000	NA
F_2–4_		0.000	0.000	NA
BC *puteoserpentis*		0.000	0.325	0.687 (13)
*B. puteoserpentis*	1.000	1.000	0.675	0.666 (27)

For the NEWHYBRIDS program the mean assignment probability (as proportion) of the chosen genotype category is given, where numbers in brackets give the sample sizes (N) that were used for averaging. *NA* Not applicable

**Fig. 4:**
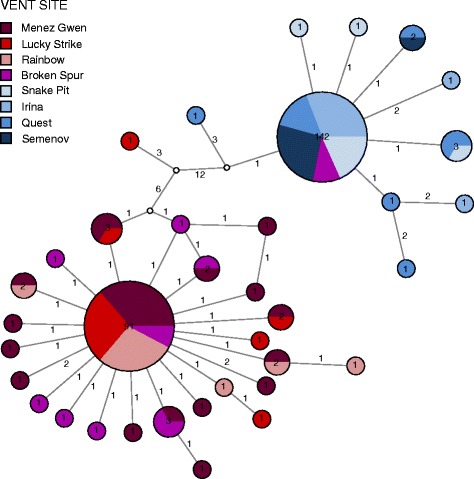
Haplotype network for the mitochondrial *ND4* locus. Haplotypes are represented as *circles*, where internal numbers indicate counts of the respective variant in the total data set and branch numbers show the mutation steps between haplotypes. Unknown variants are shown as *white circles*

Each geographic area was characterized by one major and several minor *ND4* haplotypes that formed lineage-specific clades differing by at least 12 mutations (Fig. 4). In contrast to the other localities, the BS population contained haplotypes from both lineages in roughly equal proportions (Fig. 4).

### Hardy-Weinberg equilibrium and linkage disequilibrium

Following corrections for multiple tests (BY FDR *α* = 0.0103), no significant deviations from HWE were found in any of the sampled demes. By contrast, tests for LD (BY FDR *α* = 0.0072) revealed 48 significant associations of non-homologous alleles, mostly (85.4%) involving the BS sample (Additional file 6: Table S6).

## Discussion

Studies of interspecific hybridization in animals have a long-standing history in terrestrial, coastal and shallow-water research, but virtually nothing is known about contact zones in deep-sea environments. We used 18 diagnostic SNP markers and the mitochondrial *ND4* gene to analyse the hybrid zone between the deep-sea mussels *B. azoricus* and *B. puteoserpentis* [10, 11]. With this panel of diagnostic markers we were able to reliably distinguish hybridization from incomplete lineage sorting and to estimate the extent of admixture in individuals from eight localities of the MAR. In contrast to former studies we found that evidence for introgression was widespread and not restricted to the geographically intermediate BS locality. Moreover, the frequency of hybrids was much higher than described previously (30% in [11] compared to at least 60% in this study). Gene flow occurred across the sampled geographic range from MG to SM, but in a highly asymmetric fashion. The population at BS consisted mostly of F_2–4_ generation hybrids with a minority of *B. puteoserpentis*-like genotypes, whereas no *B. azoricus*-like individuals were found. By contrast, mussels at vent fields to the north of BS carried predominantly *B. azoricus* genotypes, while mussels in the south were predominantly *B. puteoserpentis* with declining proportions of backcross genotypes. No individuals that matched an F_1_ genotype were detected at any locality. Although different statistical approaches varied in their estimates of the number of hybrids at the investigated vent locations, even the most conservative program (STRUCTURE) detected admixed individuals outside the BS hybrid zone. As many backcross genotypes were often identified as pure species and as the use of diagnostic markers might underestimate the level of introgression [27], these observations indicate that gene flow is possibly more extensive than suggested by our analyses.

Different, mutually non-exclusive explanations could account for the asymmetry in admixture patterns and the differential abundance of genotype classes among vent locations. *Bathymodiolus azoricus* and *B. puteoserpentis* appear to segregate by depth along the MAR [10, 11]. Such differential depth associations could prevent the two species from meeting and forming hybrids in certain regions. Or, if dispersal into non-native ranges occurs and the two species differ in their bathymetric tolerances, individuals with a high genomic proportion of only one parent might experience a selective disadvantage in depths that are characteristic for the other ancestral lineage. Both scenarios would agree with the differential fixation of *B. azoricus* and *B. puteoserpentis ND4* haplogroups in the northern and southern regions due to geographic premating barriers resulting in genetic drift or due to co-evolution of nuclear and cytoplasmic genomes [47, 48]. Alternatively, the apparent lack of *B. azoricus* genotypes at the intermediate BS locality could simply be a matter of sampling bias. Firstly, mussels are very difficult to distinguish from surrounding rock at this vent field as they are coated with sulphide-enriched sediments [10]. Secondly, hydrothermal vents are highly dynamic habitats, where physico-chemical conditions can vary over small spatial scales, thereby providing opportunities for niche segregation [49]. Consequently, it is possible that we failed to sample *B. azoricus* genotypes, if they are relatively infrequent or adapted to environmental patches at BS that we did not explore during our cruises. Likewise, this could be the case for F_1_ hybrids, which were surprisingly not observed in our sample. Given that we were able to detect *B. azoricus* at other localities and that the assignment certainties for F_1_ individuals were very high, it is improbable that our marker panel did not have enough power to detect these genotypes at BS. Therefore, it is justified to conclude that our results mirror a true pattern rather than a statistical artefact.

Another explanation for the observed patterns could be a southward movement of the contact zone from an unknown, possibly now extinct vent locality north of BS. Under this scenario, the early generation hybrids would be immigrants that reproduced locally. Both hybrid zone movements and potential sampling gaps would be consistent with the composition of thiotrophic symbionts in BS individuals [50]. Based on their analyses of internal transcribed spacer sequence variation, Won et al. [50] observed either single infections by the *B. puteoserpentis*-specific bacterial strain or double infections by symbionts from both species. By contrast, no single infections by the *B. azoricus*-specific type were found. Mixed infections would only be possible, when both pure species co-occur at BS (sampling gap hypothesis) or when the first contact happened at another locality (hybrid zone movement hypothesis). Both hypotheses seem to be plausible for several reasons. On the one hand, secondary contact zones between recognized species often emerge under environmental conditions to which parental genotypes are not well adapted [1, 51–53]. In such areas increased genetic diversity due to interspecific mating is likely to promote adaptability in hybrids and allow them to exploit these unoccupied niches [1]. As we did not sample environmental parameters from the investigated vent fields and were not targeting genes that might be involved in local adaptation, we cannot test this hypothesis with our current data set. However, the dynamic nature of hydrothermal vents, depths differences and the association with different chemosynthetic symbiont strains could favour ecological differentiation between *B. azoricus* and *B. puteoserpentis* and lead to hybrid advantage in environmentally intermediate vent areas. Addressing this aspect will be helpful to identify the relative importance of selective forces and neutral processes, which can cause the same introgression and admixture patterns as natural selection [54].

On the other hand, a recent study by Breusing et al. [28] indicates that hydrothermal vents or equivalent chemosynthetic habitats are much more abundant on the MAR than previously thought. In combining biophysical modelling approaches with molecular analyses the authors found that known mussel populations are connected over thousands of kilometres, although veliger larvae seldom reach suitable settlement sites that are more than 150 km away from their natal locality. These results are in agreement with current predictions from physical and geochemical measurements about the frequency of hydrothermal habitats on the MAR [55]. Thus, it is also possible that the centre of the hybrid zone originated at another, so far undetected vent locality close to BS.

Based on information from two loci (*ND4* and *Lap*), Won et al. [11] reported that the parental genotypes greatly exceeded hybrid genotypes in abundance. They hypothesized that the scarcity of mussels at BS results from a deficit of local reproduction and episodic recruitment of immigrants from the parental regions—a pattern that supported the tension zone model. The present multi-locus data cannot confirm this hypothesis, as most specimens at BS were of mixed ancestry and fell into a variety of hybrid genotype classes. Furthermore, no deviations from random mating expectations could be found, thereby providing no evidence for heterozygote deficiencies. However, the finding of significant linkage disequilibria could imply that some nuclear incompatibilities exist in hybrids. Alternatively, linkage disequilibria might be due to a recent origin of hybridization at BS, as it has been suggested that vents at this locality were lately re-activated and re-colonized after experiencing a period of dormancy [56, 57].

## Conclusions

In contrast to previous research our study shows that hybridization between *B. azoricus* and *B. puteoserpentis* is introgressive and that gene flow extends across more vent localities than reported in former work. While our results give new insights into the genetic structure of this hybrid zone, several additional analyses need to be performed to test further hypotheses on its nature and evolutionary implications. Most importantly, it will be necessary to obtain new samples from the BS vent field and to thoroughly search surrounding areas for the existence of other mussel populations. Metagenomic studies of the composition and functional variation of both thiotrophic and methanotrophic symbionts might provide critical informative data about the extent and type of ecological differentiation between parental species and hybrids. In addition, genome-wide scans could be used to design additional diagnostic markers, which—in combination with a standardized sampling design, measurements of environmental conditions and determination of phenotypic traits—would help to resolve the true degree of introgression and admixture along the MAR and the role of differential adaptation in this system. Such insights will be crucial for our general understanding of the role of hybridization in the evolution of deep-sea taxa.

## Additional files

Additional file 1: Table S1.
*Bathymodiolus* sampling localities along the Mid-Atlantic Ridge. (DOCX 17 kb)
Additional file 2:
**Table S2.** Frequencies of the *B. azoricus*-specific SNP allele in the eight sampled mussel populations; **Table S3.** SNP genotype and *ND4* haplotype data for each individual of the 8 sampled localities. 1 = c54079_g1_i1, 2 = c59751_g2_i3, 3 = c12535_g1_i1, 4 = c36135_g1_i2, 5 = c40452_g1_i1, 6 = c61080_g8_i1, 7 = c62359_g8_i4, 8 = c23539_g1_i1, 9 = c34434_g1_i1, 10 = c41151_g1_i3, 11 = c58708_g7_i1, 12 = c55170_g1_i1, 13 = c47041_g1_i1, 14 = c29533_g1_i1, 15 = c61290_g11_i1, 16 = c59181_g1_i1, 17 = c42562_g1_i1, 18 = c35975_g1_i1; **Table S4.** Fluidigm primer information (5′ to 3′) and putative gene functions of the 18 SNP loci investigated in this study. ASP1 = SNP allele detected with allele-specific primer 1, ASP2 = SNP allele detected with allele-specific primer 2, SNP_SEQ = sequence of the amplified fragment containing the SNP, ASP1_SEQ = sequence of allele-specific primer 1, ASP2_SEQ = sequence of allele-specific primer 2, LSP_SEQ = sequence of locus-specific reverse primer, STA_SEQ = sequence of forward primer for specific target amplification, AMP_GC = proportional GC content, REFSEQ = blast annotation in the RefSeq protein database, SWISSPROT = blast annotation in the Swiss-Prot database, TREMBL = blast annotation in the TrEMBL database. (XLSX 37 kb)
Additional file 3: Table S5. Genotype frequencies in the HYBRIDLAB data set as inferred by STRUCTURE, INTROGRESS and NEWHYBRIDS. For the NEWHYBRIDS program the mean assignment probability (as proportion) of the chosen genotype category is given, where numbers in brackets give the sample sizes (N) that were used for averaging. (DOCX 28 kb)
Additional file 4: Figure S1. Frequencies of hybrid and parental genotypes in the simulated population data set based on (a) STRUCTURE, (b) INTROGRESS and (c) NEWHYBRIDS. F_1_ = first generation hybrid (P_1_ x P_2_), F_2_ = second generation hybrid (F_1_ x F_1_), F_3_ = third generation hybrid (F_2_ x F_2_), F_4_ = fourth generation hybrid (F_3_ x F_3_), BC_1_ = first generation backcross (F_1_ x P), BC_2_ = second generation backcross (BC_1_ x P), BC_3_ = third generation backcross (BC_2_ x P), BC_4_ = fourth generation backcross (BC_3_ x P). (PDF 142 kb)
Additional file 5: Figure S2. Inferred genetic structure for the simulated data set for all populations based on the 18 diagnostic SNP markers. The graph confirms the existence of two mussel species that are interbreeding in an asymmetric way along the Mid-Atlantic Ridge, as shown by the presence of individuals (vertical lines) with mixed ancestry (*q*-values between 0.05 and 0.92). az = *azoricus*, pu = *puteoserpentis*, F_1_ = first generation hybrid (P_1_ x P_2_), F_2_ = second generation hybrid (F_1_ x F_1_), F_3_ = third generation hybrid (F_2_ x F_2_), F_4_ = fourth generation hybrid (F_3_ x F_3_), BC_1_ = first generation backcross (F_1_ x P), BC_2_ = second generation backcross (BC_1_ x P), BC_3_ = third generation backcross (BC_2_ x P), BC_4_ = fourth generation backcross (BC_3_ x P). (PDF 36 kb)
Additional file 6: Table S6. Significant cases of linkage disequilibrium between markers after BY FDR correction. IR = Irina, LS = Lucky Strike, QS = Quest, SP = Snake Pit, BS = Broken Spur, SM = Semenov. (DOCX 20 kb)

## Abbreviations

BC: Backcross
BS: Broken Spur
BY FDR: Benjamini-Yekutieli False Discovery Rate
*h*: Hybrid index
HWE: Hardy-Weinberg equilibrium
*IH*: Interspecific heterozygosity
IR: Irina
*Lap*: Leucine aminopeptidase
LD: Linkage disequilibrium
LS: Lucky Strike
MAR: Mid-Atlantic Ridge
MG: Menez Gwen
*ND4*: Mitochondrial NADH dehydrogenase subunit 4
QS: Quest
RB: Rainbow
SM: Semenov
SNP: Single nucleotide polymorphism
SP: Snake Pit
