# Supplementary material for: Widespread introgression in deep-sea hydrothermal vent mussels
Source: BMC Evol Biol. 2017 Jan 13;17:13. doi: 10.1186/s12862-016-0862-2 (PMC5237248; doi:10.1186/s12862-016-0862-2)
Supplement: Additional file 1: Table S1. — Bathymodiolus sampling localities along the Mid-Atlantic Ridge. (DOCX 17 kb) [file 12862_2016_862_MOESM1_ESM.docx]

**Table S1** *Bathymodiolus* sampling localities along the Mid-Atlantic Ridge

| Locality | Abbr. | Latitude | Longitude | Depth (m) | Year | Samples |
| --- | --- | --- | --- | --- | --- | --- |
| Menez Gwen | MG | 37°50.7'N | 31°31.2'W | 813–860 | 2010–2013 | 50 |
| Lucky Strike | LS | 37°17.0'N | 32°15.0'W | 1710 | 1997 | 30 |
| Rainbow | RB | 36°14.0'N | 33°54.0'W | 2251 | 1997 | 30 |
| Broken Spur | BS | 29°10.0'N | 43°10.0'W | 3350 | 1997–2001 | 30 |
| Snake Pit | SP | 23°22.0'N | 44°56.0'W | 3480 | 1997 | 30 |
| Irina | IR | 14°45.2'N | 44°58.8'W | 3020–3034 | 2007 | 48 |
| Quest | QS | 14°45.2'N | 44°58.8'W | 3024–3047 | 2007–2009 | 30 |
| Semenov | SM | 13°30.8'N | 44°57.8'W | 2432 | 2013 | 40 |
