# Supplementary material for: Widespread introgression in deep-sea hydrothermal vent mussels
Source: BMC Evol Biol. 2017 Jan 13;17:13. doi: 10.1186/s12862-016-0862-2 (PMC5237248; doi:10.1186/s12862-016-0862-2)
Supplement: Additional file 3: Table S5. — Genotype frequencies in the HYBRIDLAB data set as inferred by STRUCTURE, INTROGRESS and NEWHYBRIDS. For the NEWHYBRIDS program the mean assignment probability (as proportion) of the chosen genotype category is given, where numbers in brackets give the sample sizes (N) that were used for averaging. (DOCX 28 kb) [file 12862_2016_862_MOESM3_ESM.docx]

**Table S5** Genotype frequencies in the HYBRIDLAB data set as inferred by STRUCTURE, INTROGRESS and NEWHYBRIDS. For the NEWHYBRIDS program the mean assignment probability (as proportion) of the chosen genotype category is given, where numbers in brackets give the sample sizes (N) that were used for averaging

|  | **STRUCTURE** | **INTROGRESS** | **NEWHYBRIDS** | |
| --- | --- | --- | --- | --- |
|  | Frequency | Frequency | Frequency | Probability (N) |
| ***B. azoricus*** |  |  |  |  |
| *B. azoricus* | 1.000 | 1.000 | 0.950 | 0.690 (38) |
| BC *azoricus* | 0.000 | 0.000 | 0.050 | 0.667 (2) |
| F_1_ |  | 0.000 | 0.000 | * |
| F_2-4_ |  | 0.000 | 0.000 | * |
| BC *puteoserpentis* |  | 0.000 | 0.000 | * |
| *B. puteoserpentis* | 0.000 | 0.000 | 0.000 | * |
| **BC1 *azoricus*** |  |  |  |  |
| *B. azoricus* | 0.000 | 0.000 | 0.000 | * |
| BC *azoricus* | 1.000 | 0.675 | 1.000 | 0.991 (40) |
| F_1_ |  | 0.000 | 0.000 | * |
| F_2-4_ |  | 0.325 | 0.000 | * |
| BC *puteoserpentis* |  | 0.000 | 0.000 | * |
| *B. puteoserpentis* | 0.000 | 0.000 | 0.000 | * |
| **BC2 *azoricus*** |  |  |  |  |
| *B. azoricus* | 0.100 | 0.075 | 0.000 | * |
| BC *azoricus* | 0.900 | 0.925 | 1.000 | 0.944 (40) |
| F_1_ |  | 0.000 | 0.000 | * |
| F_2-4_ |  | 0.000 | 0.000 | * |
| BC *puteoserpentis* |  | 0.000 | 0.000 | * |
| *B. puteoserpentis* | 0.000 | 0.000 | 0.000 | * |
| **BC3 *azoricus*** |  |  |  |  |
| *B. azoricus* | 0.400 | 0.325 | 0.175 | 0.630 (7) |
| BC *azoricus* | 0.600 | 0.675 | 0.825 | 0.862 (33) |
| F_1_ |  | 0.000 | 0.000 | * |
| F_2-4_ |  | 0.000 | 0.000 | * |
| BC *puteoserpentis* |  | 0.000 | 0.000 | * |
| *B. puteoserpentis* | 0.000 | 0.000 | 0.000 | * |
| **BC4 *azoricus*** |  |  |  |  |
| *B. azoricus* | 0.700 | 0.675 | 0.400 | 0.644 (16) |
| BC *azoricus* | 0.300 | 0.325 | 0.600 | 0.772 (24) |
| F_1_ |  | 0.000 | 0.000 | * |
| F_2-4_ |  | 0.000 | 0.000 | * |
| BC *puteoserpentis* |  | 0.000 | 0.000 | * |
| *B. puteoserpentis* | 0.000 | 0.000 | 0.000 | * |
| **F_1_** |  |  |  |  |
| *B. azoricus* | 0.000 | 0.000 | 0.000 | * |
| BC *azoricus* | 1.000 | 0.000 | 0.000 | * |
| F_1_ |  | 1.000 | 1.000 | 0.997 (40) |
| F_2-4_ |  | 0.000 | 0.000 | * |
| BC *puteoserpentis* |  | 0.000 | 0.000 | * |
| *B. puteoserpentis* | 0.000 | 0.000 | 0.000 | * |
| **F_2_** |  |  |  |  |
| *B. azoricus* | 0.000 | 0.000 | 0.000 | * |
| BC *azoricus* | 1.000 | 0.000 | 0.000 | * |
| F_1_ |  | 0.000 | 0.000 | * |
| F_2-4_ |  | 1.000 | 0.975 | 0.982 (39) |
| BC *puteoserpentis* |  | 0.000 | 0.025 | 0.546 (1) |
| *B. puteoserpentis* | 0.000 | 0.000 | 0.000 | * |
| **F_3_** |  |  |  |  |
| *B. azoricus* | 0.000 | 0.000 | 0.000 | * |
| BC *azoricus* | 1.000 | 0.000 | 0.025 | 0.840 (1) |
| F_1_ |  | 0.000 | 0.000 | * |
| F_2-4_ |  | 1.000 | 0.975 | 0.964 (39) |
| BC *puteoserpentis* |  | 0.000 | 0.000 | * |
| *B. puteoserpentis* | 0.000 | 0.000 | 0.000 | * |
| **F_4_** | | | | |
| *B. azoricus* | 0.000 | 0.000 | 0.000 | * |
| BC *azoricus* | 1.000 | 0.000 | 0.000 | * |
| F_1_ |  | 0.000 | 0.000 | * |
| F_2-4_ |  | 1.000 | 0.950 | 0.984 (38) |
| BC *puteoserpentis* |  | 0.000 | 0.050 | 0.936 (2) |
| *B. puteoserpentis* | 0.000 | 0.000 | 0.000 | * |
| **BC1 *puteoserpentis*** | | | | |
| *B. azoricus* | 0.000 | 0.000 | 0.000 | * |
| BC *azoricus* | 1.000 | 0.000 | 0.000 | * |
| F_1_ |  | 0.000 | 0.000 | * |
| F_2-4_ |  | 0.775 | 0.025 | 0.979 (1) |
| BC *puteoserpentis* |  | 0.225 | 0.975 | 0.986 (39) |
| *B. puteoserpentis* | 0.000 | 0.000 | 0.000 | * |
| **BC2 *puteoserpentis*** | | | | |
| *B. azoricus* | 0.000 | 0.000 | 0.000 | * |
| BC *azoricus* | 0.875 | 0.000 | 0.000 | * |
| F_1_ |  | 0.000 | 0.000 | * |
| F_2-4_ |  | 0.200 | 0.000 | * |
| BC *puteoserpentis* |  | 0.700 | 1.000 | 0.952 (40) |
| *B. puteoserpentis* | 0.125 | 0.100 | 0.000 | * |
| **BC3 *puteoserpentis*** | | | | |
| *B. azoricus* | 0.000 | 0.000 | 0.000 | * |
| BC *azoricus* | 0.325 | 0.000 | 0.000 | * |
| F_1_ |  | 0.000 | 0.000 | * |
| F_2-4_ |  | 0.000 | 0.000 | * |
| BC *puteoserpentis* |  | 0.575 | 0.850 | 0.865 (34) |
| *B. puteoserpentis* | 0.675 | 0.425 | 0.150 | 0.610 (6) |
| **BC4 *puteoserpentis*** | | | | |
| *B. azoricus* | 0.000 | 0.000 | 0.000 | * |
| BC *azoricus* | 0.125 | 0.000 | 0.000 | * |
| F_1_ |  | 0.000 | 0.000 | * |
| F_2-4_ |  | 0.000 | 0.000 | * |
| BC *puteoserpentis* |  | 0.225 | 0.750 | 0.790 (30) |
| *B. puteoserpentis* | 0.875 | 0.775 | 0.250 | 0.622 (10) |
| ***B.* *puteoserpentis*** | | | | |
| *B. azoricus* | 0.000 | 0.000 | 0.000 | * |
| BC *azoricus* | 0.000 | 0.000 | 0.000 | * |
| F_1_ |  | 0.000 | 0.000 | * |
| F_2-4_ |  | 0.000 | 0.000 | * |
| BC *puteoserpentis* |  | 0.000 | 0.100 | 0.707 (4) |
| *B. puteoserpentis* | 1.000 | 1.000 | 0.900 | 0.639 (36) |
