# Supplementary figures and images for: Widespread introgression in deep-sea hydrothermal vent mussels
Source: BMC Evol Biol. 2017 Jan 13;17:13. doi: 10.1186/s12862-016-0862-2 (PMC5237248; doi:10.1186/s12862-016-0862-2)

(a)

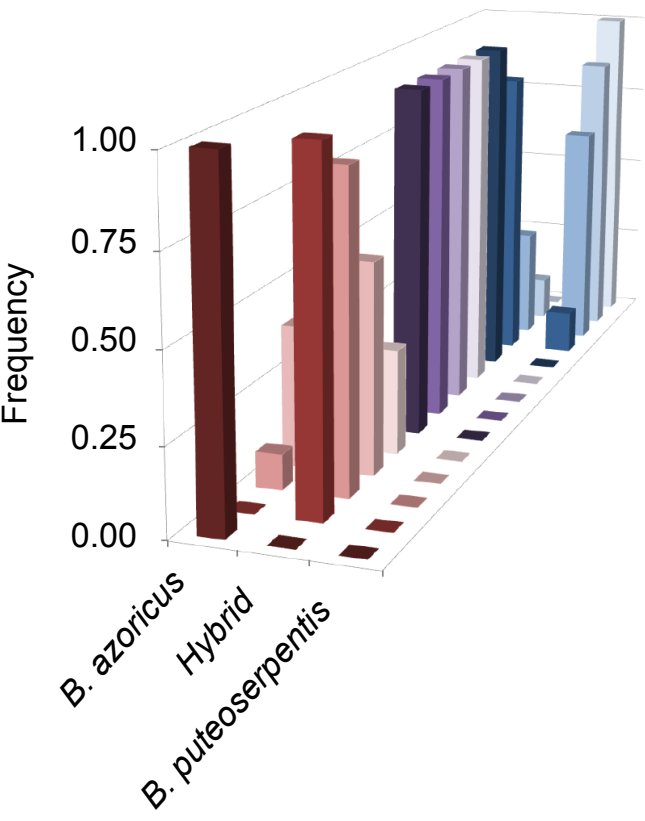

(b)

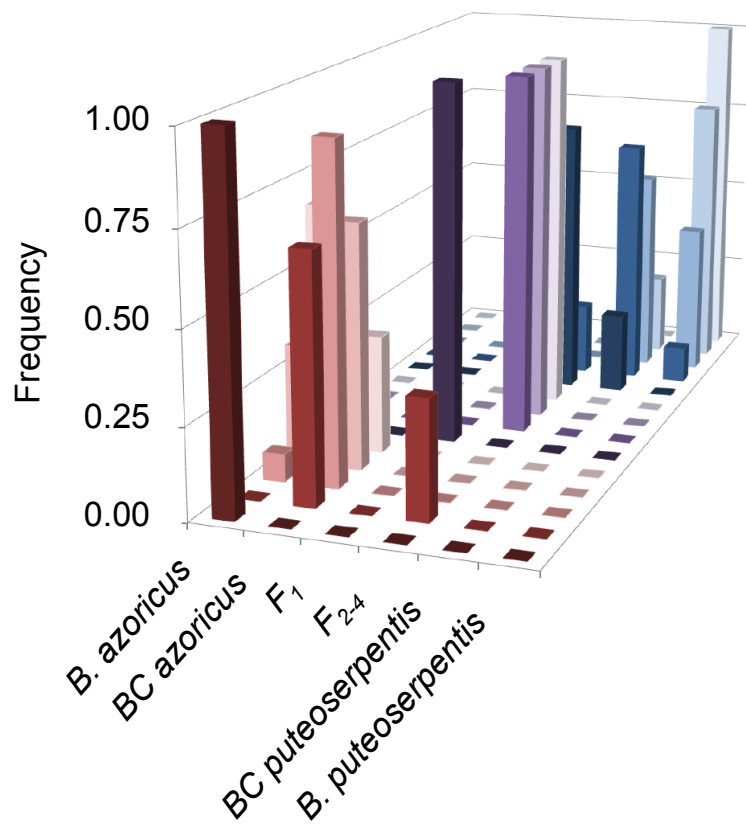

(c)

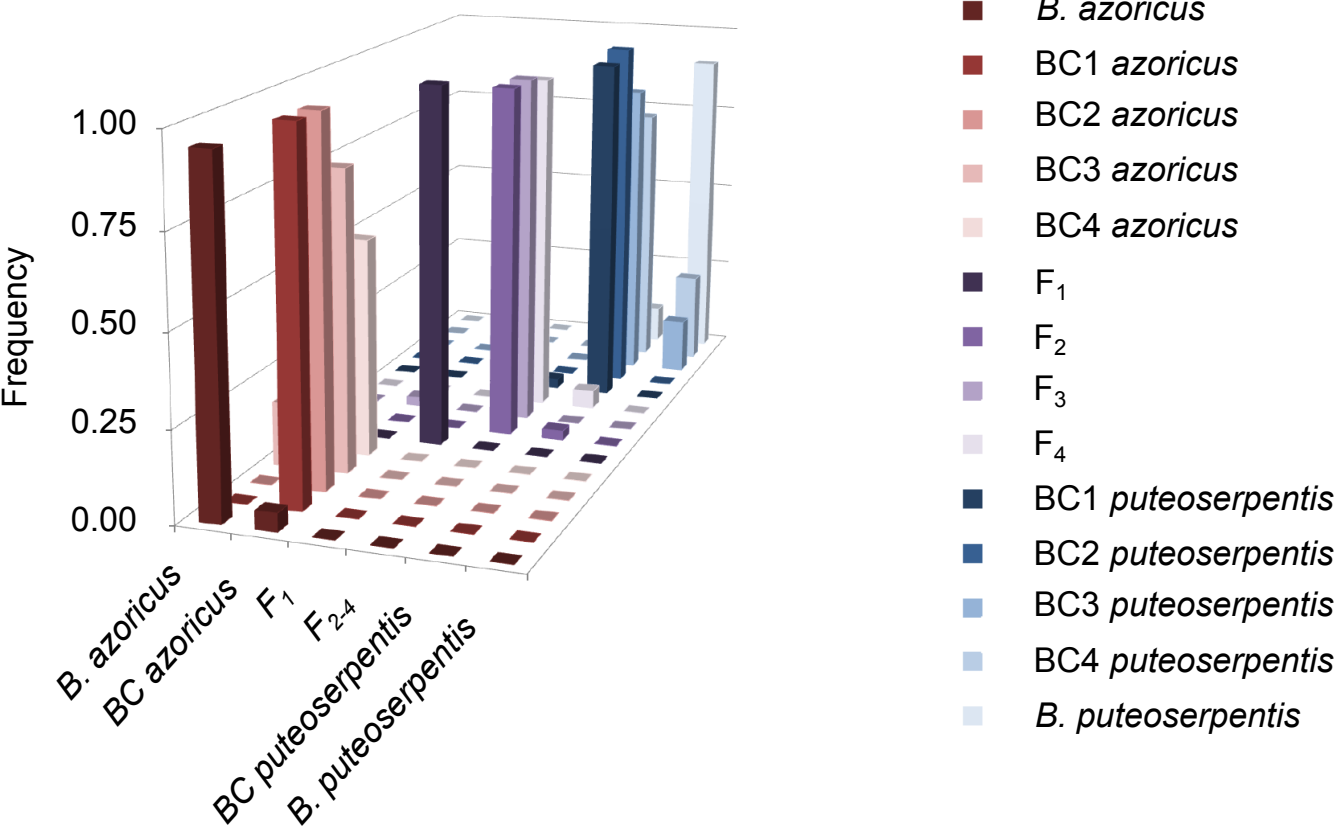

Supplement: Additional file 4: Figure S1. — Frequencies of hybrid and parental genotypes in the simulated population data set based on (a) STRUCTURE, (b) INTROGRESS and (c) NEWHYBRIDS. F1 = first generation hybrid (P1 x P2), F2 = second generation hybrid (F1 x F1), F3 = third generation hybrid (F2 x F2), F4 = fourth generation hybrid (F3 x F3), BC1 = first generation backcross (F1 x P), BC2 = second generation backcross (BC1 x P), BC3 = third generation backcross (BC2 x P), BC4 = fourth generation backcross (BC3 x P). (PDF 142 kb) [file 12862_2016_862_MOESM4_ESM.pdf]

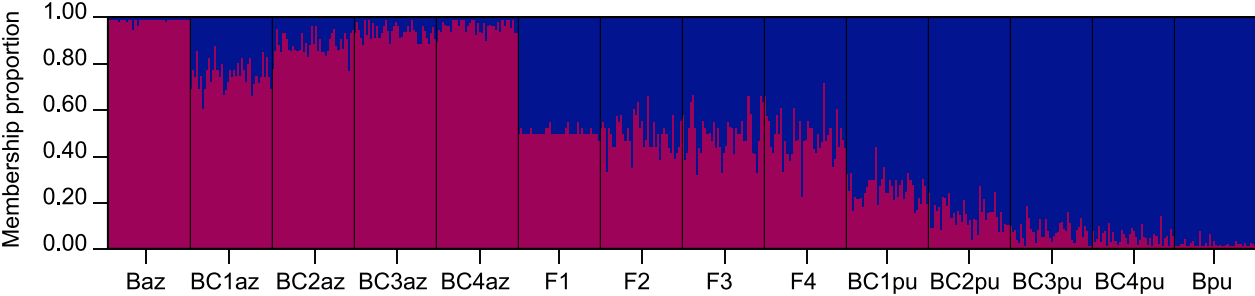

Supplement: Additional file 5: Figure S2. — Inferred genetic structure for the simulated data set for all populations based on the 18 diagnostic SNP markers. The graph confirms the existence of two mussel species that are interbreeding in an asymmetric way along the Mid-Atlantic Ridge, as shown by the presence of individuals (vertical lines) with mixed ancestry (q-values between 0.05 and 0.92). az = azoricus, pu = puteoserpentis, F1 = first generation hybrid (P1 x P2), F2 = second generation hybrid (F1 x F1), F3 = third generation hybrid (F2 x F2), F4 = fourth generation hybrid (F3 x F3), BC1 = first generation backcross (F1 x P), BC2 = second generation backcross (BC1 x P), BC3 = third generation backcross (BC2 x P), BC4 = fourth generation backcross (BC3 x P). (PDF 36 kb) [file 12862_2016_862_MOESM5_ESM.pdf]
