# Supplementary material for: Widespread introgression in deep-sea hydrothermal vent mussels
Source: BMC Evol Biol. 2017 Jan 13;17:13. doi: 10.1186/s12862-016-0862-2 (PMC5237248; doi:10.1186/s12862-016-0862-2)
Supplement: Additional file 6: Table S6. — Significant cases of linkage disequilibrium between markers after BY FDR correction. IR = Irina, LS = Lucky Strike, QS = Quest, SP = Snake Pit, BS = Broken Spur, SM = Semenov. (DOCX 20 kb) [file 12862_2016_862_MOESM6_ESM.docx]

**Table S6** Significant cases of linkage disequilibrium between markers after BY FDR correction. IR = Irina, LS = Lucky Strike, QS = Quest, SP = Snake Pit, BS = Broken Spur, SM = Semenov

| **Population** | **Gene 1** | **Gene 2** | **p value** |
| --- | --- | --- | --- |
| IR | c29533_g1_i1 | c61290_g11_i1 | 0.0000 |
|  | c58708_g7_i1 | c42562_g1_i1 | 0.0009 |
| LS | c62359_g8_i4 | c47041_g1_i1 | 0.0023 |
| QS | c29533_g1_i1 | c61290_g11_i1 | 0.0023 |
| SP | c29533_g1_i1 | c61290_g11_i1 | 0.0000 |
| BS | c62359_g8_i4 | c47041_g1_i1 | 0.0000 |
|  | c29533_g1_i1 | c61290_g11_i1 | 0.0000 |
|  | c58708_g7_i1 | c42562_g1_i1 | 0.0000 |
|  | c29533_g1_i1 | c35975_g1_i1 | 0.0000 |
|  | c61290_g11_i1 | c35975_g1_i1 | 0.0000 |
|  | c36135_g1_i2 | c35975_g1_i1 | 0.0000 |
|  | c41151_g1_i3 | c29533_g1_i1 | 0.0001 |
|  | c58708_g7_i1 | c29533_g1_i1 | 0.0001 |
|  | c62359_g8_i4 | c34434_g1_i1 | 0.0001 |
|  | c54079_g1_i1 | c62359_g8_i4 | 0.0001 |
|  | c54079_g1_i1 | c34434_g1_i1 | 0.0001 |
|  | c54079_g1_i1 | c47041_g1_i1 | 0.0001 |
|  | c58708_g7_i1 | c61290_g11_i1 | 0.0002 |
|  | c41151_g1_i3 | c61290_g11_i1 | 0.0002 |
|  | c59751_g2_i3 | c36135_g1_i2 | 0.0002 |
|  | c41151_g1_i3 | c55170_g1_i1 | 0.0002 |
|  | c12535_g1_i1 | c62359_g8_i4 | 0.0003 |
|  | c34434_g1_i1 | c61290_g11_i1 | 0.0003 |
|  | c34434_g1_i1 | c29533_g1_i1 | 0.0003 |
|  | c34434_g1_i1 | c47041_g1_i1 | 0.0005 |
|  | c12535_g1_i1 | c47041_g1_i1 | 0.0005 |
|  | c34434_g1_i1 | c35975_g1_i1 | 0.0007 |
|  | c42562_g1_i1 | c35975_g1_i1 | 0.0012 |
|  | c36135_g1_i2 | c34434_g1_i1 | 0.0015 |
|  | c36135_g1_i2 | c29533_g1_i1 | 0.0015 |
|  | c29533_g1_i1 | c42562_g1_i1 | 0.0016 |
|  | c61290_g11_i1 | c42562_g1_i1 | 0.0016 |
|  | c41151_g1_i3 | c35975_g1_i1 | 0.0021 |
|  | c23539_g1_i1 | c42562_g1_i1 | 0.0021 |
|  | c58708_g7_i1 | c35975_g1_i1 | 0.0022 |
|  | c41151_g1_i3 | c42562_g1_i1 | 0.0024 |
|  | c36135_g1_i2 | c62359_g8_i4 | 0.0035 |
|  | c59751_g2_i3 | c29533_g1_i1 | 0.0038 |
|  | c41151_g1_i3 | c58708_g7_i1 | 0.0041 |
|  | c36135_g1_i2 | c61290_g11_i1 | 0.0042 |
|  | c54079_g1_i1 | c12535_g1_i1 | 0.0043 |
|  | c54079_g1_i1 | c59181_g1_i1 | 0.0054 |
|  | c54079_g1_i1 | c55170_g1_i1 | 0.0055 |
|  | c55170_g1_i1 | c29533_g1_i1 | 0.0057 |
|  | c54079_g1_i1 | c35975_g1_i1 | 0.0063 |
|  | c62359_g8_i4 | c42562_g1_i1 | 0.0066 |
| SM | c29533_g1_i1 | c61290_g11_i1 | 0.0013 |
|  | c62359_g8_i4 | c47041_g1_i1 | 0.0013 |
